# Supplementary material for: Multiple Long-Term Conditions (MLTC) and the Environment: A Scoping Review
Source: Int J Environ Res Public Health. 2022 Sep 13;19(18):11492. doi: 10.3390/ijerph191811492 (PMC9517156; doi:10.3390/ijerph191811492)
Supplement: Supplementary file 1 [file ijerph-19-11492-s001.zip › ijerph-1878497-Supplementary.pdf]

Supplementary Materials

Table S1. Key characteristics of included studies.

Study	Source—First Author and Date	Setting (e.g., Primary Care, Secondary Care)	Country (e.g., UK, USA)	Population Characteristics (e.g., Age, Sex, Ethnicity and Disease Group)	Sample Size	Study Type (Qualitative, Mixed Methods or Quantitative)	Study Aims	Findings (Including Links with MLTC)	Environmental Determinants Discussed
I	Wu, Yu-Tzu, 2020. The longitudinal associations between proximity to local grocery shops and functional ability in the very old living with and without multimorbidity: Results from the Newcastle 85+ study.	Community/neighbourhood setting.	UK.	People aged 85+.<br><br>People with and without MLTC.<br><br>Males and Females.<br><br>No ethnicity specified.	A population-based cohort of 852 people.	Quantitative.	To identify environmental factors that support maintenance of functional ability, the aim of this study is to investigate the longitudinal associations between proximity to local grocery shops and the ability to shop for groceries in the very old and to examine the potential variation between those living with and without multimorbidity.	The very old who lived in more deprived areas were more likely to have a grocery shop within 500 m than those in less deprived areas. Proximity to local grocery shops had limited impacts on those who were relatively healthy (0–1 chronic condition), but moderated loss of the ability over time in those living with multimorbidity. Proximity to local grocery shops was, however, found to support the very	'Other environmental factors (safety, traffic, stairs, the quality of pavement, age-friendliness of local shops) … might affect use of local shops.'

								old living with multimorbidity and their ability to do grocery shopping.	
II	Adhikari, B. 2021. Community design and hypertension: Walkability and park access relationships with cardiovascular health.	Community / neighbourhood setting.	Canada.	Two independent population cohorts of adults.<br><br>Hypertension and related cardiovascular disease (CVD).<br><br>The studies recruited participants using different age limits, the average age of participants was 45.6 years and 55.1 years for MHMC and BC Gen, respectively.<br><br>Ethnicity was categorized into five groups: (i) Caucasian, (ii) Aboriginal, (iii) Asian, (iv) South Asian, and (v) for MHMC and into two groups: (i) Caucasian and (ii) others for BC Gen.	One Cohort was 22,418 adults (My Health My Community) and the other cohort was 11,972 adults (BC Generations Project).	Quantitative.	To examine how features of the built and natural environments relate to hypertension and the mediating role of transportation and leisure walking and body mass index in this relationship.	Findings demonstrate that living in a walkable neighbourhood and having higher park accessibility is associated with lower odds of hypertension, especially for lower income individuals. We suggest an integrated population health approach that considers multimorbidity as a result of exposure to car-dependent areas and the lack of green spaces.	Neighbourhood walkability and park accessibility may help mitigate adverse health consequences of hypertension by reducing the number of individuals with body mass index.

**III**	Alkhatib, A. 2021. Preventing Multimorbidity with Lifestyle Interventions in Sub-Saharan Africa: A New Challenge for Public Health in Low and Middle-Income Countries.	No specific setting.	Sub-Saharan Africa.	Adults aged 18+.<br><br>Males and females.<br><br>Multimorbidity clusters, especially hypertension, diabetes and cardiovascular disease could provide an early effective prevention of multimorbidity in LMICs [Low and Middle Income Countries].	Not applicabl e.	Narrative review.	This narrative review summarises key epidemiological multimorbidity determinants and highlights specific public health challenges related to multimorbidity prevention strategies, especially through lifestyle in LMICs placing emphasis on Sub-Saharan Africa, and its future regionally relevant health policies.	LMICs [Low and Middle Income Countries] are experiencing a fast-paced epidemiological transition towards multimorbidity, characterized by clusters of NCDs [Non-communicable Diseases], which require public health interventions. Multimorbidity determinants include increased age, female sex, environment, lower socio-economic status, obesity, and lifestyle behaviours, especially poor nutrition, physical inactivity.	Long term negative impact of environmental factors on health outcomes has particularly focused on air pollution associations with the development of chronic asthma, pulmonary insufficiency, CVD [cardiovascular disease], cancer, and diabetes.
**IV**	Adamkiewicz. G. 2014. Environmental Conditions in Low-	Low-income housing developments.	USA.	Participants from the adult population of 20 publicly and privately	828	Mixed methods.	We explored prevalence and clustering of key	Environmental problems were common; more	Mold, combustion by-products,

	Income Urban Housing: Clustering and Associations With Self-Reported Health.			managed low-income housing developments across 3 cities (Cambridge, Somerville, and Chelsea) in the metropolitan area. of Boston, Massachusetts.<br><br>Mainly females but some males.<br><br>Residents of the developments were eligible for participation if they were aged 18 years or older and spoke English, Haitian Creole, or Spanish.			environmental conditions in low-income housing and associations with self-reported health.	than half of homes had 3 or more exposure-related problems. After adjustment for household-level demographics, we found clustering of problems in site for pests, combustion by products, mold, and ventilation.	second-hand smoke, chemicals, pests, and inadequate ventilation.
**V**	Hernandez-Garcia, E. 2021. Research capacity strengthening in health and care delivery through housing for chronic diseases.	No specific setting.	No geograp-hy specified.	No specific population specified.<br><br>No age range specified. No ethnicity specified.	Not applicabl e.	Systematic review.	The aim was to determine relevant evidence gaps to building capacity in supportive housing-health care research about chronic diseases and multimorbidity, identify ongoing	Future research needs to prioritise articulating these emerging insights through systematic, translational and multisectoral approaches to strengthen health and care delivery through housing	Impact of housing design on MLTC.

							strains and offer potential solutions.	design solutions for people living with chronic morbidity.	
**VI**	Adamkiewicz, G. 2014. Neighborhood Environment and Type 2 Diabetes Comorbidity in Serious Mental Illness.	Community/Neighbourhood setting.	Australia.	Adults over 18 years old with a "Serious Mental Illness" such as schizophrenia, bipolar disorder, or major depression.<br><br>No ethnicity specified.<br><br>Males and Females.	3816.	Quantitative.	We aimed to investigate the associations of neighborhood environments with T2D comorbidity in individuals with SMI.	T2D comorbidity in SMI is a major public health issue.<br>We observed that individuals with SMI residing in areas with higher crime rates were more likely to report T2D comorbidity compared to individuals with SMI residing in lower crime rate areas, even after controlling for individual-level variables and neighborhood-level disadvantage.<br>Overall, the study suggests that the mechanisms of neighborhood influence on SMI-	Our study focused on 5 neighborhood-level variables**:** (1) neighborhood-level crime, (2) access to health care services, (3) neighborhood-level obesity, (4) availability of green spaces, and (5) availability of fast-food outlets.

								T2D are highly complex.	
**VII**	Ou, J. Y. 2018. Self-rated health and its association with perceived environmental hazards, the social environment, and cultural stressors in an environmental justice population.	Community / People at home.	USA.	Adults over 18 years of age.<br><br>Hispanic urban population.<br><br>Predominantly female sample.	354.	Mixed methods.	We examined self-rated health and its association with multiple types of perceived environmental hazards in a majority-Hispanic urban population.	We identify strong associations among fair/poor self-rated health and participant-reported environmental hazards, the social environment, and cultural stressors. We confirm the roles of environmental, social, and cultural stressors on self-rated health even after adjusting for chronic and mental health conditions. Our findings shed light on the complex sources of chronic stress in an environmental justice community, and their impacts on the health of a majority-Latino population.	Self-rated health, perceptions of their neighborhood, including participant-reported environmental hazards (e.g., air quality, odors and noise), aspects of the social environment (e.g., feeling safe, neighborhood crime, social cohesion), and culture-related stressors (e.g., immigration status, language stress, ethnic identity).

**VIII**	Villena, A.L.D. 2010. Challenges and struggles: lived experiences of individuals with co-occurring disorders.	Community/Neighbourhood setting Community treatment centers and supportive housing sites.	USA.	Mean age 51.<br><br>60% with MLTC.<br><br>40% with one condition.<br><br>Psychiatric and physical diseases.<br><br>Participant characteristics: 11 men and 9 women; (65% African American).	20.	Mixed methods Qualitative interviews. Quantitative analysis of conditions and demographics.	The purpose of this interpretive study was to understand, describe, and illustrate the social and structural barriers that individuals with COD [Co-occurring disorders] of mental illness, substance abuse, and general medical conditions encounter in regard to their health care.	Living with COD was perceived to be a “constant struggle and challenge” by informants. Their primary and pervasive complaint was that relationships with health care providers served as impasses to managing health. Informants felt misunderstood and at times voiceless. In addition, inadequate insurance coverage and medication capitations created additional burdens for this population trying to manage multiple health problems simultaneously. These barriers were worsened by	Navigating healthcare system. Trust with providers (doctors). Quality of accommodation/housing including stability. Support from family and friends.

								living in contexts that made health management difficult, such as unkempt and dangerous housing accommodations.	
**IX**	Ingram, E. 2020. Household and area-level social determinants of multimorbidity: a systematic review.	Community/neighbourhood.	North America, Europe and Austral-asia.	Participants from the general population and assessed for the presence of multiple chronic conditions (multimorbidity).<br><br>Participants solely young people (age < 18 years) excluded._Age range, mainly those aged 50 and over.<br><br>Thirty-six of 41 studies included a mix of chronic physical and mental health conditions, while four included physical conditions only.<br><br>Males and Females.<br><br>No ethnicity specified.	Not applicable.	Systematic review.	This review aimed to systematically identify, critically appraise and synthesise the existing literature on associations between household and area-level Social Determinants of Health and multimorbidity prevalence or incidence. In general populations of high-income countries (HICs). We also aimed to investigate how associations differ with age, gender and ethnicity.	Those living in the most deprived areas had the highest prevalence or incidence of multimorbidity. Associations between deprivation and multimorbidity differed by age and multimorbidity type. Findings from the few studies investigating household tenure, household composition and area-level rurality were mixed and contradictory; homeownership	Household tenure, household composition and area-level rurality. Trust in neighbours (n=1): One fairly low-quality study found that participants who somewhat distrusted their neighbours had increased risk of developing multimorbidity within 11 years compared with those who strongly disagreed with

							Better understanding of SD of multimorbidity could inform equitable prevention and intervention strategies.	and rurality were associated with increased and decreased multimorbidity, while living alone was found to be associated with a higher risk of multimorbidity and not associated.	the statement 'One cannot trust each other here' (RR 1.13, 95% CI 1.03 to 1.23).
**X**	Olufunke A. 2013. The social determinants of multimorbidity in South Africa.	Community/People at home.	South Africa.	Adults over 18 years of age.<br><br>Males and Females. Surveys were given to all above 15 but those below 18 were removed.<br><br>A diverse range of ethnic groups in a South African context.	11,638.	Quantitative.	The aim of this study was to determine the prevalence of multimorbidity and examine its association with various social determinants of health in South Africa.	Social capital (defined as 'how people connect with others in their environment'), was not associated with multimorbidity or even health per se.	Social capital.
**XI**	Health behaviors and multimorbidity resilience among older adults using the CanadianlStudy on Aging.	Community/People at home.	Canada.	Canadian adults aged 65 or older who reported two or more of 27 chronic conditions.<br><br>Conditions included: Alzheimer's disease, back problems, bowel	6,771.	Quantitative.	This study examines associations between lifestyle behavioral factors and multimorbidity resilience (MR)	All four of the social/environmental variables exhibited statistically significant, albeit weak, associations with multimorbidity.	Social and environmental variables: number of friends, number of relatives, housing problems and

				incontinence, cancer, cataracts, diabetes, epilepsy, glaucoma, heart attack, heart disease, high blood pressure, irritable bowel syndrome, kidney disease, Parkinson’s disease, peripheral vascular disease, lung disease, macular degeneration, multiple sclerosis, osteoarthritis, osteoporosis, migraine headaches, rheumatoid arthritis, stroke, thyroid problem, transient ischemic attack, ulcer, and urinary incontinence.<br><br>No ethnicity specified.<br><br>Males and Females.			among older adults.		rural/urban status.
**XII**	Speldewinde, P.C. 2011; The hidden health burden of environmental degradation: disease comorbidities and dryland salinity.	Community (rural).	Australia.	The case populations with the three diseases (asthma, suicide and ischaemic heart disease) studied between 1996 and 2001, were extracted from	Total populati on of the study area averaged 408,111.	Quantitative.	Given the emerging evidence of the effects of environmental degradation on mental health, it was hypothesised	Using georeferenced health record data, Bayesian spatial methods were used to determine the relationship	The effects on health of environmental degradation related to dry salinity.

	Western Australia's Data Linkage Unit database. Indigenous people.<br><br>Adults 18+.<br><br>Males and Females.		that typically comorbid diseases may also be influenced by environmental degradation. In Western Australia, a recent study (Speldewinde et al., 2009) found an association between dryland salinity and depression. This rural population was re-examined for a range of other physical and psychological conditions—particularly asthma, ischaemic heat disease and suicide—to identify whether any corresponding increased risk was associated with the presence of a depressive illness.	between dryland salinity and a range of human health outcomes. Initial modelling found an increased relative risk for asthma, suicide and ischaemic heart disease in relation to dryland salinity (adjusted for Indigenous and socio-economic status). However, in this follow-up study, a further evaluation of the role of co-morbidities in this population revealed that: (i) the presence of depression was consistently linked to residence in areas with high salinity and (ii) the association of asthma, suicide and heart disease with salinity was

	most likely attributable to the co-morbidity of the conditions with depression.	
	Total included	12
